# A112 VIDEO CAPSULE ENDOSCOPY REGISTRY OF ONTARIO (VCERO) - RETROSPECTIVE PHASE (NORTHERN ONTARIO REGION)

**DOI:** 10.1093/jcag/gwab049.111

**Published:** 2022-02-21

**Authors:** C Lavalle, M Yaghoobi, D Armstrong, K Tilbe

**Affiliations:** 1 Western University Schulich School of Medicine & Dentistry, London, ON, Canada; 2 Northern Ontario School of Medicine - East Campus, Sudbury, ON, Canada; 3 McMaster University, Hamilton, ON, Canada

## Abstract

**Aims:**

The primary aim of this project is to determine the feasibility of establishing and maintaining a registry of all video capsule endoscopy procedures performed at a large regional center. Secondary aims include the determination of indications for capsule endoscopy, quality measures, and procedural outcomes at a large regional center and the comparison of these metrics to other locations globally.

**Methods:**

A retrospective collection and analysis of all video capsule endoscopies performed at Health Sciences North in Sudbury Ontario between 2016 and 2020 has been performed. Information was collected with the aid of a standardized data collection tool and stored under anonymized patient profiles. Once this pilot registry was created, data was pooled for further analysis. Data regarding indications for capsule endoscopy, patient factors, previous investigations, anticoagulant usage, procedure interpretation, need for subsequent interventions, and need for blood transfusion among others was then established for this group. Findings were then compared to previously published regional and global norms where available.

**Results:**

In total 98 video capsule endoscopies encompassing 94 individual patients have been catalogued at the end of the study period. With available information through individual patient records, complete information for all fields of the standardized collection tool was obtained for 91 individual patients. The most common indications for VCE include gastro-intestinal bleeding (38%), anemia (38%), Crohn’s disease (13%) and abdominal pain (3%). In total, 45% of all procedures noted an acute finding in the report. Of VCE performed for gastro-intestinal bleeding, 50% reported on an acute finding while in those performed for anemia, 47% reported on an acute finding. Mortality during the study period was 12% while there was 1 documented incident of capsule retention. The rate of acute findings is greater than previously published international studies.

**Conclusions:**

This project has established the feasibility of the creation of a regional capsule endoscopy registry. Further work to expand the patient pool as well as move to a prospective collection model are reasonable next steps to expand this project. We have also demonstrated significant differences regarding indications for VCE as well as the rate of significant findings of VCE of a our northern cohort compared to previously reported international sites. In particular the high rate of significant findings in anemia warrants further investigations.

**Funding Agencies:**

None

